# Free-standing plasmonic metal-dielectric-metal bandpass filter with high transmission efficiency

**DOI:** 10.1038/s41598-017-04540-9

**Published:** 2017-06-28

**Authors:** Yuzhang Liang, Si Zhang, Xun Cao, Yanqing Lu, Ting Xu

**Affiliations:** 1 National Laboratory of Solid State Microstructures, College of Engineering and Applied Sciences and Collaborative Innovation Center of Advanced Microstructures, Nanjing University, 22 Hankou Road, Nanjing, 210093 China; 2 School of Electronic Science and Engineering, Nanjing University, 22 Hankou Road, Nanjing, 210093 China

## Abstract

Plasmonic spectrum filtering devices based on metallic nanostructures have attracted wide attention due to their good reliability, ease of fabrication, and wideband tunability. However, the presence of thick substrate significantly limits the structure’s longitudinal size for further optoelectronic integration and reduces the devices’ performance. Here we propose and demonstrate an ultra-thin plasmonic bandpass filter based on free-standing periodic metal-dielectric-metal stack geometry working in the near-infrared wavelength range. The coupling between free-space electromagnetic waves and spatially confined plasmonic modes in the designed structure is systematically investigated. As demonstrated in the calculation and experiment, the free-standing plasmonic filters have more than 90% transmission efficiency and superior angular tolerance. The experimental results are in good agreement with the theoretical calculations. These artificial nanostructured filtering devices may find potential applications in the extremely compact device architectures.

## Introduction

In the past few years, surface plasmons (SP) in the metallic nanostructures have attracted a tremendous amount of attentions due to its unique and outstanding properties for manipulation of light^1–5^. One of the most conspicuous applications for metallic nanostructures is spectrum filtering^6–12^. With the development of nanofabrication and characterization techniques, various plasmonic filters have been proposed and experimentally demonstrated. For example, plasmonic filters with periodic holes or slit array in a single layer metal film can obtain high transmission efficiency based on the extraordinary optical transmission effect^13–17^, and usually are 1–2 orders of magnitude thinner in comparison to conventional pigment-based filters. However, most of the single layer plasmonic filters are very sensitive to the incident angle, which is not suitable for the applications that require a large field of view. To overcome the drawbacks of poor angular tolerance, an effective approach is to employ the coupling between two metallic layers to design the filter^18–22^. In addition, almost all reported plasmonic filters are incorporating thick substrate, which not only severely restricts the device’s critical thickness and functionality in the ultra-compact integrated optical systems, but also reduces the device’s performance due to asymmetric coupling occurring between the top and bottom interfaces^23–26^. Recently, bandpass filters based on single layer metal grating integrated with transparent membranes have been implemented at mid-infrared frequencies^27–29^. Although these devices have very small thickness, the transmission efficiencies are still limited due to the devices’ asymmetric geometry.

In this paper, we propose and demonstrate an ultra-thin near-infrared filter with high transmission efficiency and superior angular tolerance. The structure design is based on a free-standing periodic metal-dielectric-metal (MDM) stack geometry. Compared with above-mentioned devices, the filter’s thickness is remarkably reduced due to the absence of transparent substrate. The absence of thick substrate and symmetric MDM geometry make the designed filter achieve high transmission efficiency over 90% and two-fold enhancement of the angular tolerance. Furthermore, the introducing of the additional slit pattern is able to significantly reduce the bandwidth of transmission peak. This design concept can also be applied to other wavelength ranges.

## Results and Discussions

### Plasmonic bandpass filter based on free-standing MDM stack array

**Figure 1:**
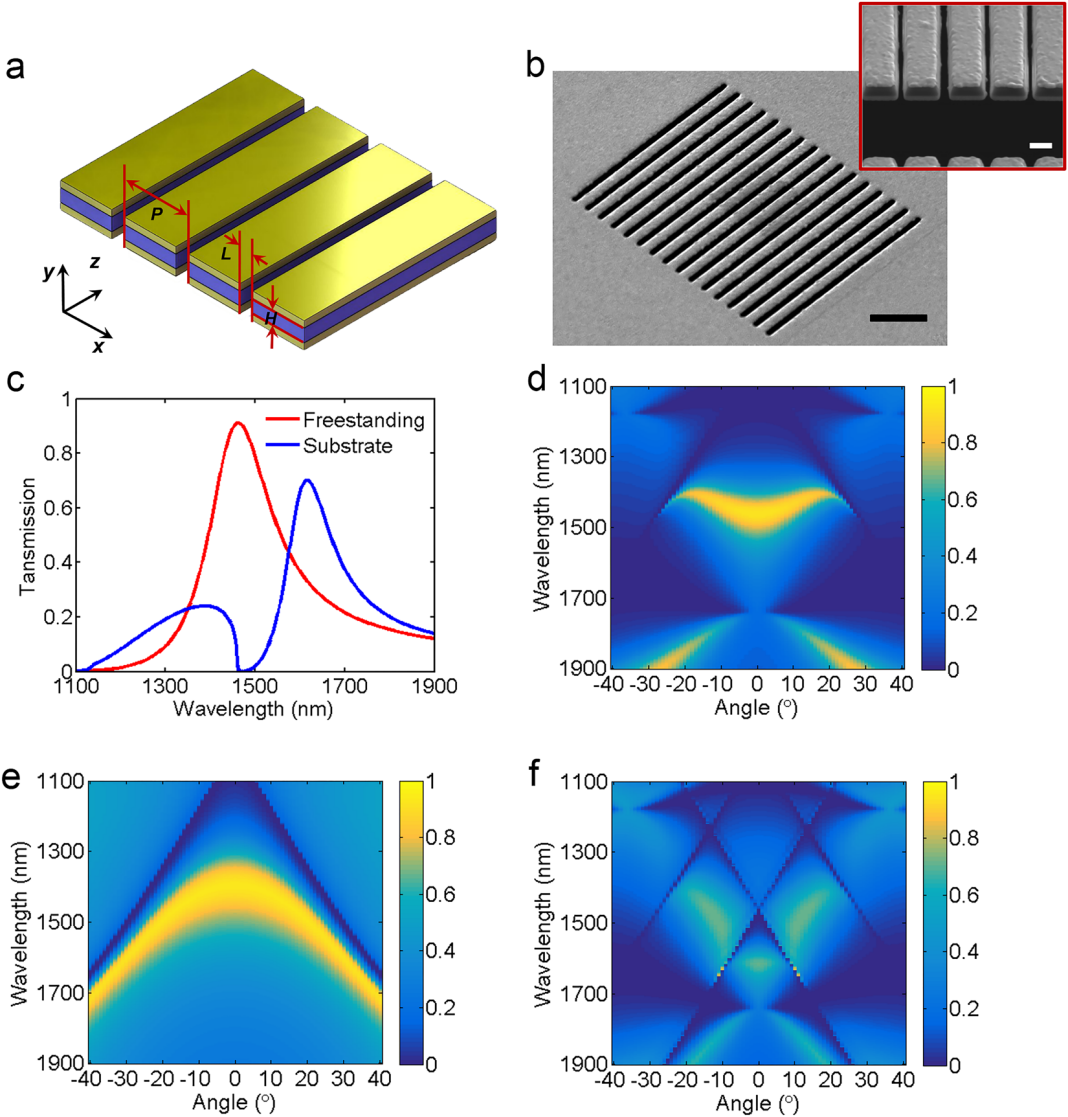
Plasmonic bandpass filter constructed by free-standing MDM stack array. (**a**) Schematic diagram of the free-standing MDM stack array. The Si_3_N_4_ dielectric layer is sandwiched in between Au layers. The periodicity (*P*) and slit width (*L*) of the array is 1000 nm and 250 nm. The thickness of Si_3_N_4_ dielectric layer and Au layers is 250 nm and 90 nm, respectively. (**b**) Scanning electron microscopy images of the fabricated device. Scale bar, 4 μm. Inset shows magnified cross-section view. Scale bar, 500 nm. (**c**) Comparison of calculated transmission spectra for the free-standing structure and the structure with substrate. Calculated transmission diagrams as a function of incident angle and wavelength for (**d**) the free-standing MDM stack array, (**e**) the free-standing single layer metallic grating, and (**f**) the MDM stack array with substrate.

Figure 1a presents the schematic diagram of the proposed MDM stack array, which has periodic narrow slits all the way through the Au/Si_3_N_4_/Au film. The structure is designed to act as a bandpass filter in the telecom wavelength range with structural parameters as following: periodicity *P = *1000 nm, slit width *L* = 250 nm, the thickness of waveguide layer *H* and Au film are 250 nm and 90 nm, respectively. For TM polarized incident light (the *E* -field is perpendicular to the Au grating direction), it can be coupled into SP mode through the top Au grating to the waveguide layer, then bottom Au grating reconverts the confined plasmon to propagating waves again and transmits the incident light to far field. Here the 250-nm-thick waveguide layer is optimized to ensure the efficient coupling of SP waves on the top and bottom surfaces of waveguide layer and the 90-nm-thick Au layer can prohibit the direct transmission of incident light. On the other side, for TE polarized light (the *E* -field is parallel to the Au grating direction), it cannot be coupled into SP mode by the top Au grating and thus are almost totally reflected by the structure.

The designed free-standing MDM stack array is fabricated by depositing 90-nm-thick Au film both on the top and bottom side of a 250-nm-thick Si_3_N_4_ membrane supported by a Si substrate. The structure is patterned using focused ion beam (FIB) over a 15 μm × 15 μm area. Figure 1b shows the scanning electron microscopy (SEM) image of the fabricated sample. A cross-sectional SEM image in the inset of Fig. 1b clearly shows the fabricated free-standing trilayer Au/Si_3_N_4_/Au stack array. To demonstrate the advantages of free-standing structure, the transmission spectrum of the structure is first investigated by using the full-wave simulations based on the finite-difference time-domain (FDTD) algorithm (see Methods). Figure 1c shows the calculated transmission spectra of MDM stack array with (solid blue line) and without substrate (solid red line). There are three main differences between two spectra: First, compared to that of the free-standing structure, the wavelength λ_R_ of transmission peak of the substrate structure has an obvious redshift. In other words, to achieve the same transmission peak, array periodicity of substrate structure is smaller than that of free-standing structure, which increases the difficulty of experiment preparation. Second, for substrate structure, there is another small transmission peak in the wavelength range from 1100 nm to 1460 nm as the by-product of structure design, which is resulted from asymmetric coupling from the substrate. Last but not least, compared with the transmission efficiency about 70% from the substrate structure, the free-standing structure has more than 90% transmission efficiency at the peak.

Besides high transmission efficiency, the free-standing MDM stack array also shows a good angular tolerance. Figure 1d presents the angle-resolved transmission spectra for free-standing MDM stack array. When the incident angle increases from 0° to 20°, the transmission peak always keeps high transmission efficiency and the wavelength of transmission peak changes from 1462 nm to 1408 nm. The angular tolerance of transmission peak is defined as the wavelength shift respect to incident angle changes. The smaller angular tolerance is better. Therefore, the angular tolerance of transmission peak is 2.7 nm per degree. As comparisons, here we also calculate the angle-resolved transmission spectra for the free-standing single layer metallic grating structure (Fig. 1e) and the MDM stack array with substrate (Fig. 1f). It can be clearly seen that for the free-standing single layer metallic grating structure, although the transmission efficiency is also high in the incident angle range from 0° to 20°, the angular tolerance of transmission peak is much worse (5.4 nm per degree). For the MDM stack array with substrate, the efficiency of transmission peak is lower and the range of angular tolerance is narrower (about from −5° to 5°) because of asymmetric coupling and the appearance of new transmission dips and peaks from the substrate. Compared to the above two structures, the free-standing MDM stack array has a superior angular tolerance and keeps high transmission efficiency, which is very important for the development of practical devices.

**Figure 2:**
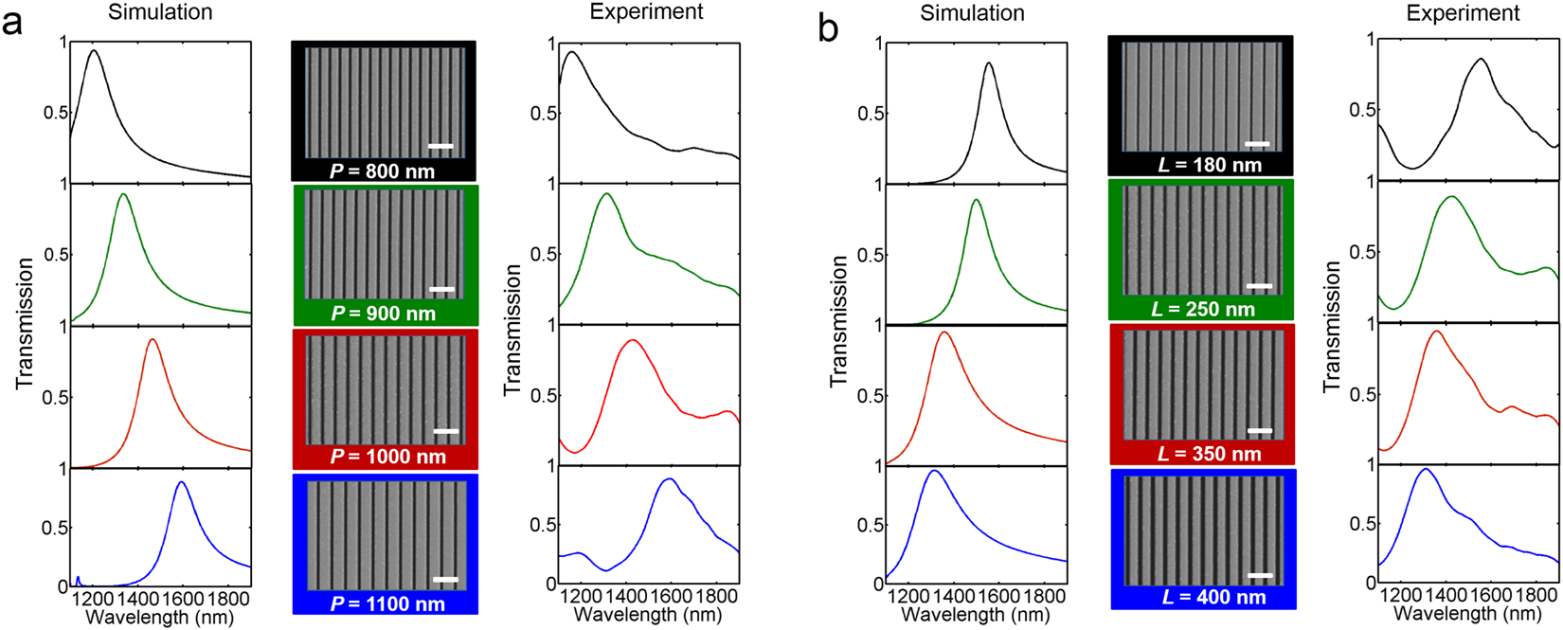
Calculated and measured transmission spectra for various periodicity *P* and slit width *L*. (**a**) Periodicity ***P*** from top to bottom are 800, 900, 1000, 1100 nm. (**b**) Slit width ***L*** from top to bottom are 180, 250, 350, 400 nm. In Fig. 2(a) and (b), left column is calculated from FDTD simulations, and right column is from experiment. Central column shows the corresponding SEM images. Scale bar, 2 μm.

To investigate the dependence of wavelength position of transmission peak on periodicity *P* and slit width *L*, the transmission spectra with different *P* and *L* are depicted in Fig. 2. The left column in Fig. 2a and b is calculated from the numerical simulations (see Methods), while the right column shows the experimental results. We can see that the efficiency of transmission peak is more than 90% in the designed free-standing structure. The wavelength of transmission peak exhibits a redshift with the structure periodicity ranging from 800 to 1100 nm with fixed slit width *L* = 250 nm, and has a blue-shift as slit width *L* increases from 180 nm to 400 nm with fixed *P* = 1000 nm. The wavelength position of transmission peak has a good agreement between numerical simulations and experiment. Compare to the calculated transmission spectra, the experimental curves in Fig. 2 have some additional transmission peaks at long wavelength range or short wavelength range, and transmission peaks have wider bandwidth. This is because theoretical curves are calculated under normal incidence. However, in the experiment, a lens with a numerical aperture (NA) of 0.2 is used for focusing the incident light into sample surface, which means that our experiment curves are cumulative data from incident angle range from 0° to 12°. As shown in Fig. 1(d), we can see clearly that when incident angle increases gradually, the additional peaks appear at the long wavelength range or short wavelength range. These peaks would disappear under normal incidence.

**Figure 3:**
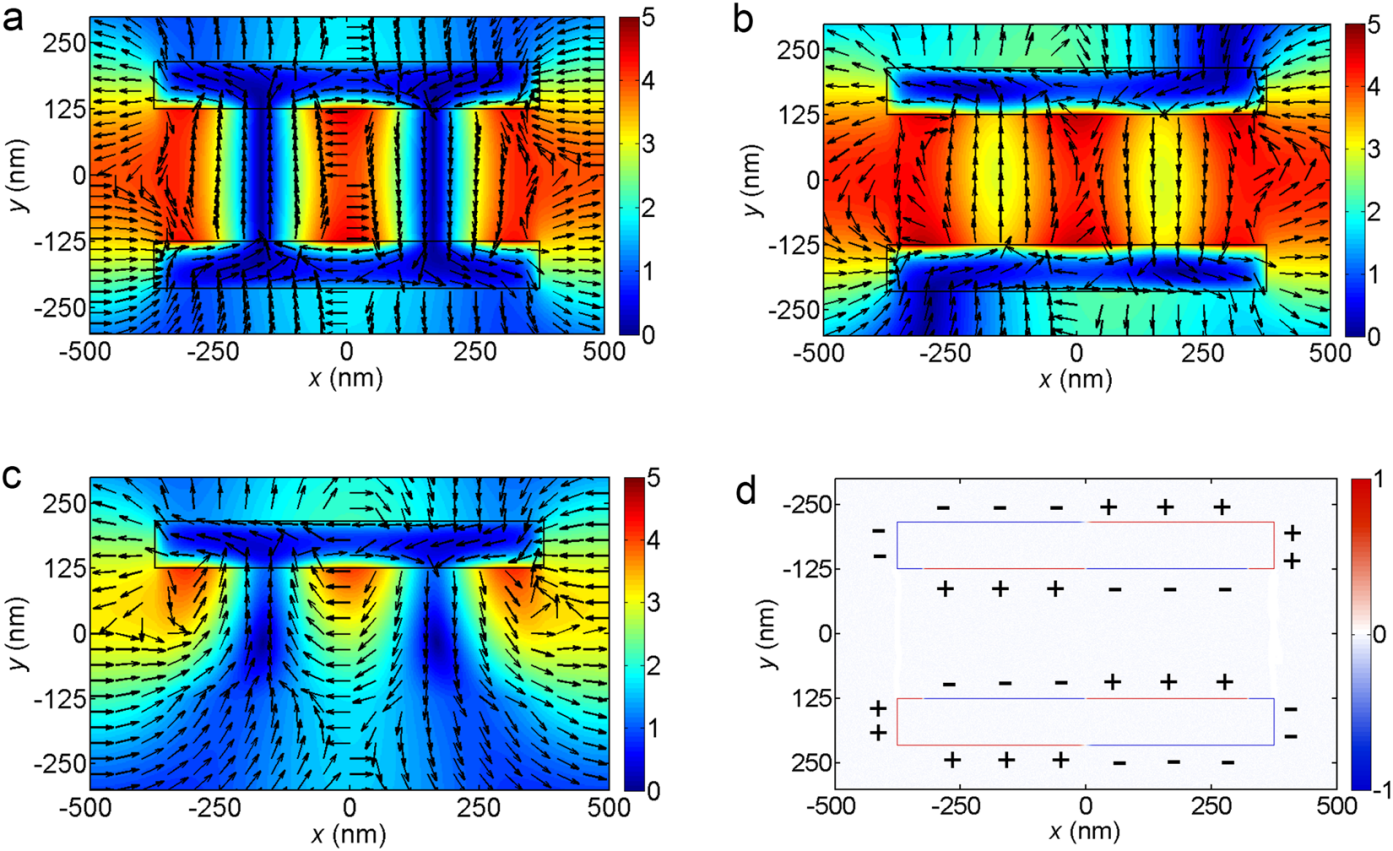
Plasmonic modes in the free-standing MDM stack array and single layer metallic grating. (**a**) Calculated magnetic field intensity |*H*_z_| (color map), electric displacement (arrow map), and (**d**) charge distribution at the transmission peak 1462 nm under normal incidence. (**b**) Calculated magnetic field intensity |*H*_z_| and electric displacement at the transmission peak 1412 nm with incident angle 15°. (**c**) Calculated magnetic field intensity |*H*_z_| and electric displacement at the transmission peak 1398 nm under normal incidence. (**a**,**b**,**d**) free-standing MDM stack array, (**c**) free-standing single metallic grating.

In order to better explain the origin of spectrum filtering effect, the normalized magnetic field intensity (color map) and electric displacement (arrow map) in one unit-cell corresponding to the wavelength of 1462 nm under normal incidence are shown in Fig. 3a. The periodicity *P* and slit width *L* is 1000 nm and 250 nm, respectively. The magnetic field intensity shows that most of incident energy is localized both within the MDM waveguide layer and the air slit. On one hand, inside the MDM waveguide layer, the antisymmetric waveguide modes is formed along the *x* direction^30^, which is characterized by maximum magnetic intensity near the edges of top and bottom Au grating. Electric displacement represented by the black arrows in the top and bottom metallic gratings are opposite to each other and forms a loop, which generates a significant magnetic response. So MDM waveguide modes along *x* direction can be also considered as the magnetic dipole. The excitation of magnetic dipole in the designed structure increases the capacity of angular tolerance^31^. Figure 3b depicts corresponding electromagnetic field distribution at the wavelength 1412 nm with incident angle 15°. Although incident angle increases from 0° to 15°, MDM waveguide mode along the *x* direction remains nearly the same. However, for the free-standing metallic grating, significant magnetic response cannot be formed (Fig. 2c), which results in poor angular tolerance. On the other hand, the antisymmetric mode in the air slit is formed along the *y* direction. Compared to that in the MDM waveguide layer, this is a truncated mode. Here, the antisymmetric mode in the air slit is named as the cavity mode to distinguish that of MDM waveguide layer. In addition, this mode in the air slit can be considered as a Fabry-Perot cavity that possesses two mirrors of finite reflection at the end of the slit. Due to the symmetry structure with respect to a vertical mirror, the cavity mode bisects the middle section of slit^32–35^. The formation of cavity mode in the air slit leads to high transmission. The main difference of above two resonant modes is the direction of propagation, and the interaction of these two modes results in the generation of transmission peak in the free-standing MDM stack array. Figure 3d shows the charge distribution in one unit-cell corresponding to the wavelength of 1462 nm. The charge distribution along *x* direction of MDM waveguide layer and *y* direction of air slit is nearly identical, which further verifies the existence of two antisymmetric modes in the structure.

Furthermore, the shift of transmission peak in Fig. 2 can be explained qualitatively by using waveguide mode along the *x* direction. For the case with fixed air slit width but increasing the stack periodicity, the effective permittivity of the waveguide layer in MDM stack array increases, resulting in a redshift of the transmission wavelength based on three-layer waveguide theory^36^. On the other side, for the case with fixed stack periodicity but increasing air slit width, the effective permittivity of the waveguide layer in MDM stack array decreases, resulting in a blue-shift of the transmission wavelength.

### Narrow-band plasmonic filter based on the free-standing dual-slit MDM stack array

**Figure 4:**
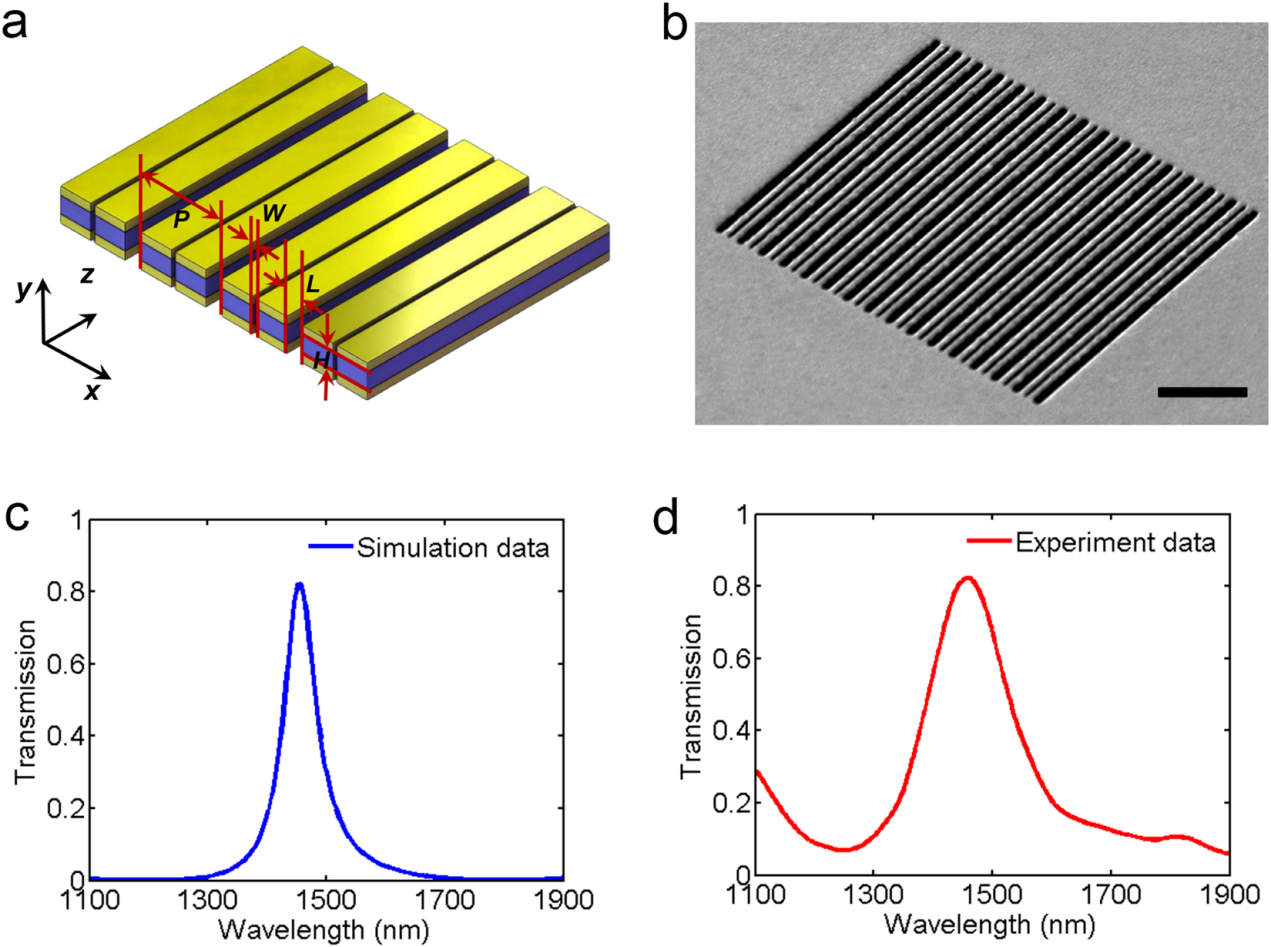
Narrow-band plasmonic bandpass filter constructed by free-standing dual-slit MDM stack array. (**a**) Schematic diagram of the free-standing dual-slit MDM stack array. The dual-slit array is formed by piercing periodic narrow slits of width *W* = 80 nm on the structure of Fig. 1(a). Other structure parameters are identical to that in Fig. 1(a). (**b**) SEM images of the fabricated dual-slit device. Scale bar, 4 μm. (**c**) Calculated and (**d**) measured transmission spectra for the free-standing dual-slit MDM stack array.

For above designed MDM structure, it is difficult to obtain a narrow transmission peak just by changing structural parameters. To circumvent this problem, here we present a dual-slit MDM stack geometry, as shown in Fig. 4a. This structure can be fabricated by piercing another set of narrow slits all the way through the MDM stack film. Figure 4b shows the oblique view SEM image of the fabricated dual-slit MDM stack array. The periodicity *P* and slit width *L* of the MDM array is 1000 nm and 250 nm. The width of second slit *W* in the middle of MDM stack is 80 nm. Calculated transmission spectrum of the dual-slit structure is depicted in Fig. 4c. The transmission peak has a ~64 nm full width at half maximum (FWHM), which is narrower than ~156 nm FWHM of transmission peak in the single-slit MDM structure. Compared to that of single-slit MDM structure, the efficiency of transmission peak for the dual-slit MDM structure is a little lower but still more than 80%. Figure 4d shows experimentally measured transmission spectrum of the dual-slit structure. The FWHM of transmission peak is ~150 nm, wider than that of the numerical simulations, but narrower than experiment result of single-slit structure (~241 nm) shown in Fig. 2. The difference between numerical simulations and experiment results can be attributed to three reasons: First, the dielectric permittivity of Au and Si_3_N_4_ layers used in the numerical simulations has a little different from that in experiment. Second, there are the inevitable roughness of structure surface and imperfect fabrication process. Third, the microscope objective with a non-zero numerical aperture in the experiment measurement brings oblique incidence, widening the line shape of transmission peak and introducing additional transmission peaks.

**Figure 5:**
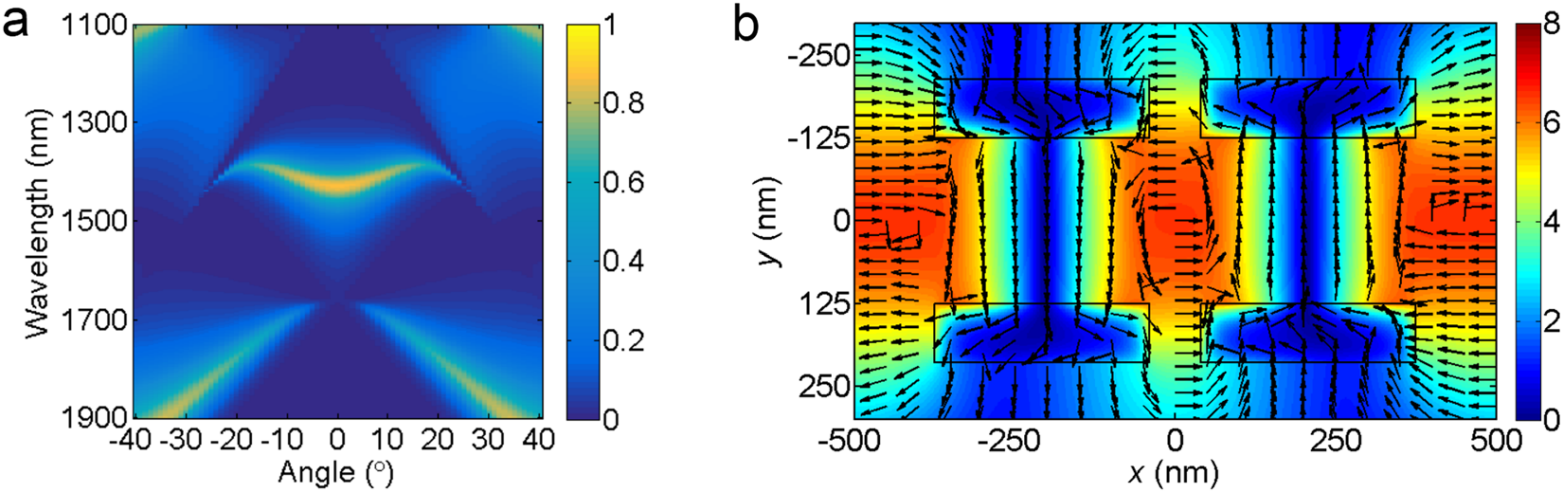
(**a**) Calculated transmission diagrams as a function of incident angle and wavelength in the dual-slit structure. (**b**) Calculated magnetic field intensity |*H*_z_| (color map) and electric displacement (arrow map) at the transmittance peak 1454 nm for the dual-slit structure.

Figure 5a presents transmission spectra under different incident angles in the dual-slit MDM structure. The wavelength position 1454 nm of transmission has a little change and keeps high transmission efficiency in the incident angle ranging from −20° to 20°. It is noteworthy that the transmission peak of dual-slit MDM structure keeps narrow bandwidth. Due to similarity between Fig. 5a and Fig. 1d, we infer that the physical mechanism of transmission peak in the dual-slit structure is nearly identical to that of single-slit structure. The calculated electromagnetic field distribution at the transmission peak shown in Fig. 5b verifies above viewpoint. The electromagnetic field distribution in Fig. 5b is almost identical to that in Fig. 3a. The only difference is that second slit affecting the antisymmetric mode of MDM stack array and decreasing the effective permittivity of the waveguide layer, which is the reason why the wavelength of transmission peak has a small blue-shift in comparison to that of single-slit MDM structure.

**Figure 6:**
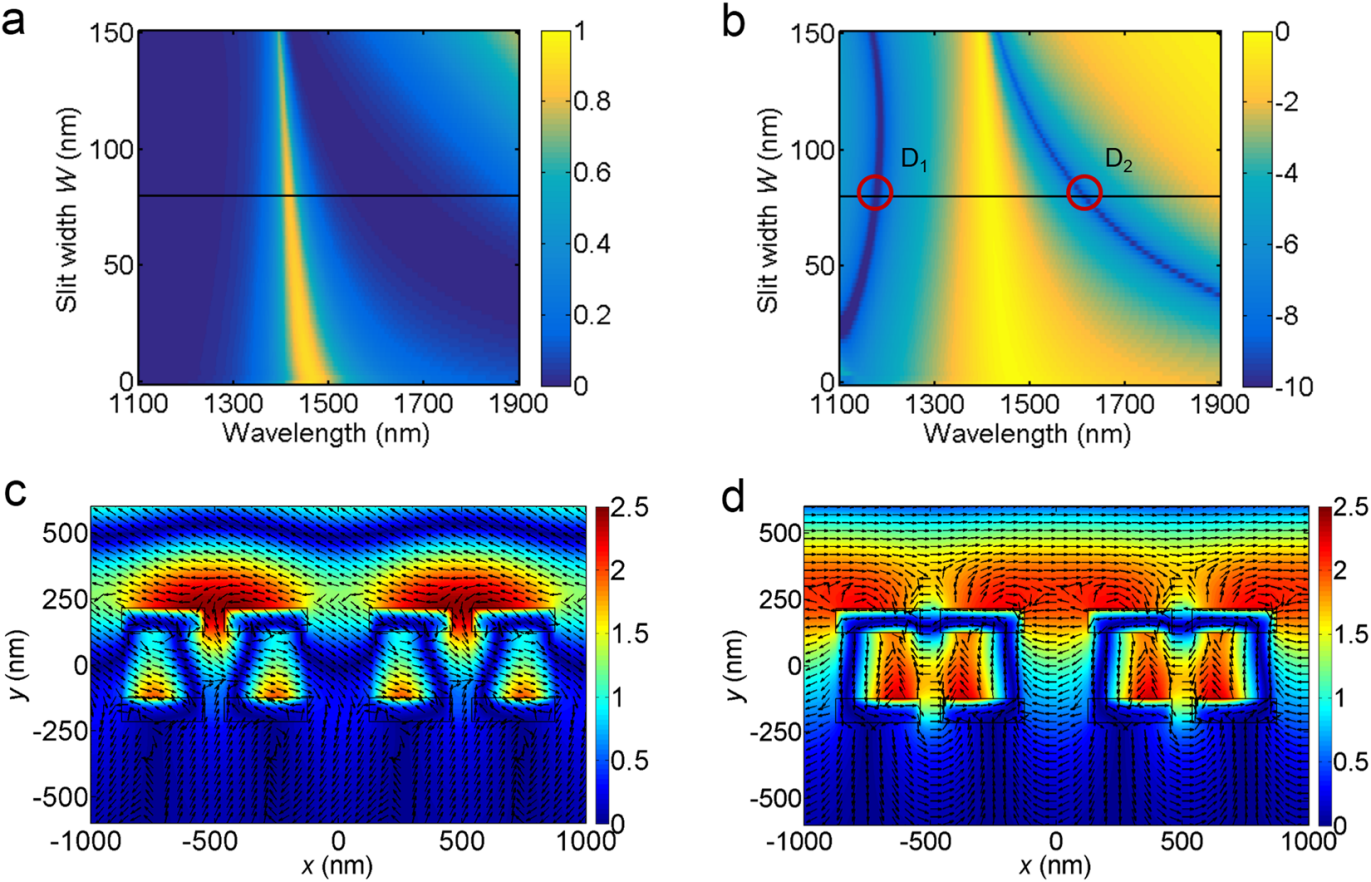
Calculated transmission in (**a**) linear color map (**b**) logarithmic color map as a function of slit width *W* and wavelength. The red circles D_1_ and D_2_ correspond to two transmission minima with slit width *W* = 80 nm. Calculated magnetic field intensity |*H*_z_| (color map) and electric displacement (arrow map) for (**c**) D_1_ and (d) D_2_.

Next, we perform the numerical simulations to investigate the relationship between the slit width *W* and the transmission spectrum, as shown in Fig. 6a. We find that there are two transmission minimum strips in the wavelength range from 1100 to 1900 nm when slit width *W* is larger than 35 nm. To better illustrate these transmission minimum strips, the results in Fig. 6a are converted into logarithmic scale and shown in Fig. 6b. Clearly, as the slit width *W* increases, the transmission minimum strip at longer wavelength has an obvious blue-shift while the transmission minimum strip at shorter wavelength doesn’t change much, which makes the bandwidth of transmission peak become narrower. Therefore, the appearance and shift of the transmission minimum strip is the reason for narrowing the transmission bandwidth in the dual-slit structure. Furthermore, as shown in theoretical curve of Fig. 2b, as the slit width decreases, bandwidth of transmission peak reduces gradually. Dual-slit MDM structure integrates two sets of single slit structures with different slit width. Compared to that of single-slit MDM structure, dual-slit MDM structure possesses the spectral characteristics of the structure with narrow slit width. Therefore, dual-slit array could provider a narrow bandwidth than the single slit case. This is another reason for narrow bandwidth of transmission peak for dual-slit MDM structure.

In order to investigate the physical origin of the generation of transmission minimum strips, the calculated magnetic field intensity (color map) and electric displacement (arrow map) with slit width *W* = 80 nm are given in Figs. 6c and 6d, respectively, corresponding to the transmission minimum wavelengths D_1_ and D_2_ shown in Fig. 6b. At the transmission minimum wavelength D_1_, we can see that the magnetic field is mainly localized on the upper surface of the metallic grating which is attributed to the excitation of SP on the upper surface induced by grating structure. The SP excitation condition of the metallic grating is defined as^37, 38^

1 $$\frac{2\pi }{\lambda }\,\sin \,\theta -n\frac{2\pi }{P}=-\frac{2\pi }{\lambda }\sqrt{\frac{{\varepsilon }_{m}{\varepsilon }_{d}}{{\varepsilon }_{m}+{\varepsilon }_{d}}}={k}_{spp}\quad \quad n=0,\pm 1,\pm 2,\mathrm{...},\pm N$$

where λ is the incident light wavelength, *n* is diffraction order, *θ* is the incident angle, *P* is the grating periodicity, and *ε*_d_ and *ε*_m_ stand for the permittivity of dielectric and metal. On the upper surface of structure, there is a wave crest for magnetic field distribution in one unite cell, corresponding to 1st order of SP mode (n = 1 in Eq. 1). However, the existence of additional slit makes the localized electromagnetic field on the upper surface of structure leak into MDM waveguide layer, which results in the excitation of 2nd order SP mode on the upper surface of bottom Au grating, where there are two wave crests for magnetic field distribution in one unite cell. The SP modes propagating along the two upper surfaces of the Au grating eventually reflects the incident light in the backward direction, which reduces the transmission close to zero.

At the transmission minimum wavelength D_2_, we can see that strong magnetic fields are localized in MDM waveguide layer and electric displacement forms a loop, which means that antisymmetric waveguide mode is formed along the *x* direction in the MDM waveguide layer. Different from the single-slit MDM structure shown in Fig. 3a, here the SP modes inside the MDM structure induced by the second slit would interfere with the cavity mode. At the transmission minimum wavelength D_2_, the destructive interference occurs at the slit exit and so that significantly suppresses the optical transmission.

## Conclusions

High performance and ultracompact size are two important features for the next generation photonic components. In the previous reports^23–26^, the existence of structure substrate not only degrades device’s performance, but also remains the longitudinal thickness of device in the range of a few hundreds of micrometers, which significantly limits device’s integration. Here, our designed plasmonic device has a longitudinal thickness a few hundreds of nanometers, 2–3 orders of magnitude thinner than that of reported filters, which is very attractive for the design of ultracompact integrated optoelectronics system.

In summary, we propose and experimentally demonstrate a new type of ultra-thin plasmonic bandpass filter based on the free-standing MDM stack array. Compared with conventional plasmonic devices, this filter has higher transmission efficiency and better angular tolerance. Moreover, the bandwidth of transmission peak can be further tuned by introducing the dual-slit geometry. Due to its good spectral filtering performance, the proposed device is a potential candidate for developing a high performance photonic platform for near-infrared spectral imaging system. The design principle can also be easily expanded to other wavelength ranges for multispectral applications.

## Methods

### Numerical simulations

The simulations are performed by using a commercial software (FDTD solutions, Lumerical Solutions) based on FDTD algorithm to obtain the transmission spectra, magnetic field distribution and electric displacement distribution of the free-standing MDM stack array. At the normal incidence, the periodic boundary conditions are applied in *x* direction and perfectly matched layers are used in *z* direction. However, the bloch boundary conditions need to be applied in *x* direction under condition of oblique incidence. The grid size in the *x* and y direction is 1 × 1 nm, respectively, in order to satisfy the integer number of grid in simulation region. The dielectric permittivity of bulk gold in the near-infrared region is from Johnson and Christy^39^, and the refractive index of the Si_3_N_4_ waveguide layer is 2. All the sizes are set according to the measured ones from the SEM images.

### The preparation and measurement of free-standing MDM stack array

Structures are fabricated on a commercially available 250-nm-thick Si_3_N_4_ membrane with a 3 × 3 mm window opened in the silicon layer. A 3-nm Ti film and a 90-nm Au film are e-beam evaporated sequentially onto the front and back sides, respectively. The deposition rate for Ti and Au is R_Ti_ ≈ 0.016 nm s^−1^ and R_Au_ ≈ 0.02 nm s^−1^, respectively. Then, subwavelength gratings are fabricated by FIB milling using a dual-beam (FIB/SEM) system (Ga^+^ ions, 24 pA beam current, 30 k eV beam energy). To prevent the Au from water in the air, we store the samples in a nitrogen gas holder before measurement. All transmission spectra are measured by using a commercial microspectrometer (PV20/30 from CRAIC Technologies). A lens with a numerical aperture about 0.2 is used for focusing the incident light onto the sample surface. Transmission light is collected using a 20× microscope objective with a numerical aperture of 0.45. Measurement area is set to an area of roughly 13 × 13 μm^2^.
